# Quality of Health Management Information System for Maternal & Child Health Care in Haryana State, India

**DOI:** 10.1371/journal.pone.0148449

**Published:** 2016-02-12

**Authors:** Atul Sharma, Saroj Kumar Rana, Shankar Prinja, Rajesh Kumar

**Affiliations:** School of Public Health, Post Graduate Institute of Medical Education and Research, Chandigarh, India; University College London, UNITED KINGDOM

## Abstract

**Background:**

Despite increasing importance being laid on use of routine data for decision making in India, it has frequently been reported to be riddled with problems. Evidence suggests lack of quality in the health management information system (HMIS), however there is no robust analysis to assess the extent of its inaccuracy. We aim to bridge this gap in evidence by assessing the extent of completeness and quality of HMIS in Haryana state of India.

**Methods:**

Data on utilization of key maternal and child health (MCH) services were collected using a cross-sectional household survey from 4807 women in 209 Sub-Centre (SC) areas across all 21 districts of Haryana state. Information for same services was also recorded from HMIS records maintained by auxiliary nurse midwives (ANMs) at SCs to check under- or over-recording (Level 1 discordance). Data on utilisation of MCH services from SC ANM records, for a subset of the total women covered in the household survey, were also collected and compared with monthly reports submitted by ANMs to assess over-reporting while report preparation (Level 2 discordance) to paint the complete picture for quality and completeness of routine HMIS.

**Results:**

Completeness of ANM records for various MCH services ranged from 73% for DPT1 vaccination dates to 94.6% for dates of delivery. Average completeness level for information recorded in HMIS was 88.5%. Extent of Level 1 discordance for iron-folic acid (IFA) supplementation, 3 or more ante-natal care (ANC) visits and 2 Tetanus toxoid (TT) injections was 41%, 16% and 2% respectively. In 48.2% cases, respondents from community as well as HMIS records reported at least one post-natal care (PNC) home visit by ANM. Extent of Level 2 discordance ranged from 1.6% to 6%. These figures were highest for number of women who completed IFA supplementation, contraceptive intra-uterine device insertion and provision of 2nd TT injection during ANC.

**Conclusions:**

HMIS records for MCH services at sub-centre level in Haryana state were satisfactory in terms of completeness. However, there were significant differences in terms of reported and evaluated coverage of MCH services. Quality of HMIS needs to be improved in order to make it relevant for public health program planning and research.

## Introduction

World Health Organisation (WHO) in 2007 identified Health Management Information System (HMIS) as a key building block of health system.[1] In India, the emphasis has been laid on creation and utilization of an effective HMIS system under the National Rural Health Mission (NRHM), flagship healthcare program under the Ministry of Health and Family Welfare. Since NRHM’s inception in 2005, several mechanisms have been put in place to improvise and strengthen its functioning. One of these efforts includes revision and simplification of existing HMIS to avoid duplication. Secondly, a national web-based HMIS portal was set-up in 2008 for collection and sharing of data in a timely manner.[2] The focus of these activities under National Rural Health Mission was to emphasize upon local planning and use of evidence during decision making.

An effective HMIS not only serves to monitor the performance and quality of the health services being provided but also provides a sound evidence platform to base decisions upon, by acting as a repository of information for various healthcare indicators collecting data from the community and healthcare providers.[3] Utilisation of routine MIS is not limited to policy makers and program managers. Researchers also draw upon it to answer critical health system questions pertaining to effectiveness or efficiency of health programs. Numerous such efforts have been carried out recently in India.[4, 5] But an HMIS is only successful to the extent to which it ensures production, analysis, dissemination and use of information reliably and in a timely fashion.[6]

Despite its unequivocal importance for routine decisions, HMIS has repeatedly been reported to be riddled with many problems.[7] Not only have the records suffered incompleteness and poor quality, there is a tendency to over-report the outputs and outcomes.[3, 8] Irregularities in reports generation, data duplication and data inconsistencies, at all levels of healthcare delivery extending from SCs to district and state level, are commonly observed and reported. However there is no robust analysis to assess the HMIS in India. We aim to bridge this gap in evidence base by assessing the extent of completeness and quality of HMIS in Haryana state.

## Methodology

### Study Setting and Conceptual Framework

Haryana state, with a population of 25 million, has been divided into 21 administrative regions known as districts. The public health system is a vertical 3-tier machinery, with a SC over every 5000 population at the grassroot level. SC staff comprise of Auxillary Nurse Midwives (ANMs) and a male Multipurpose Health Worker (MPHW), who are provided with five record registers, through which they maintain information on reproductive health, child health and basic preventive & curative health care services delivered at facility. Every SC submits a report on number of beneficiaries who received these services in last calendar month to the next level i.e. Primary Health Centre (PHC). Five to six SCs are monitored by a PHC which caters to 30000 population.

Conceptually, there are two points in the chain at which quality of data can be influenced. The first point is the entry of beneficiary information and provision of services in registers at SC (Level 1 discordance). The second point of over-reporting potentially happens at the time of reporting to higher facilities (Level 2 discordance). This means that differences may be observed between the actual coverage of maternal and child health services in the community and information being recorded in ANM registers, or between the latter and the periodic reports being submitted by ANMs to the PHC. Both these aspects need to be taken into account for making a comprehensive statement on the quality and accuracy of the HMIS. For this purpose, information for indicators needs to be collected from three sources: actual coverage from community, ANM record registers and monthly reports submitted by ANMs to PHC.

### Study design

For information from the community, data was extracted from a larger cross-sectional household survey being currently undertaken in Haryana state as part of the Concurrent Evaluation of National Health Mission.[9] Under this survey, thirty graduate field investigators trained in data collection interview a range of beneficiaries in randomly selected SC areas. These beneficiaries include women who had delivered a child in 1 year preceding the survey, children between 12 to 23 months of age and eligible couples. A total of 23 women were selected in each SC to assess coverage of MCH services received during antenatal, delivery and postnatal period. This constituted dataset 1 for our study.

For level 1 discordance, eight MCH data elements were selected from the larger survey. (Table 1) These were selected after weighing criteria like importance of the data element, probability of recall bias in answers and inclusion of element in HMIS record registers maintained by ANMs. Details of data elements and subsequently generated indicators can be found in S1 Table. After completion of data collection from the respondents in the field in a particular SC area, the field investigators collected data from SC records on these eight elements for all 23 respondents. This was done for 209 SC areas (4807 women included in dataset 1) who had delivered a baby during last year preceding the survey. This formed dataset 2.

**Table 1:** Data elements for assessing quality of health management information system in Haryana state, India.

**Mismatch between survey and Sub-Centre record (Level 1 discordance)**
Number of ANC Check-ups received by pregnant woman during ANC
Number of TT injections received by pregnant woman during ANC
Number of IFA tablets received by pregnant woman during ANC
Place of delivery: Public sector health facility, private health facility or home delivery
Date of delivery
Outcome of pregnancy: Live birth or still birth
Number of PNC home visits conducted by ANM
DPT 1 Vaccination Date
**Mismatch between Sub-Centre record and Sub-Centre report (Level 2 discordance)**
Number of pregnant women registered during last month
Number of pregnant women who received TT1 dose
Number of pregnant women who received TT2 dose
Number of pregnant women who were initiated on prophylactic IFA tablets course
Number of pregnant women who were initiated on therapeutic IFA tablets course
Number of pregnant women who completed prophylactic IFA tablets consumption course
Number of pregnant women who completed therapeutic IFA tablets consumption course
Number of women who delivered a child in public sector health facility/ private facility/ home
Number of women who delivered a child in private health facility
Number of women who delivered a child at home
Number of DPT1/ LPV1 vaccines administered
Number of Measles 1 vaccines administered
Number of Vitamin A doses administered
Number of women registered under SC who received IUD insertions

To evaluate level 2 discordance in HMIS, i.e. record to report, a separate data collection tool was prepared to compare data in ANM records with that in reports sent to PHCs. Fourteen data elements, available in both sources, were selected for inclusion in this tool. (Table 1) As submitted monthly reports contain cumulative figures for the previous month and not individual data, hence data for selected fourteen elements were calculated by totalling individual records for last month from registers by the field investigators. This constituted dataset 3. Finally, figures for the same elements were obtained from last month’s report submitted by the facility to state headquarter. This formed our dataset 4. Dataset 3 and 4 were generated from a random sub-sample of 89 SC facilities.

To assess the quality of data collection by field investigators during the period of data collection supervisory visits were undertaken by the research team. A total of 61 health sub-centre areas (29% of total) were covered under this supervision exercise, where 667 women (13.8% of the total) were interviewed again by the supervisor to collect data on the MCH service utilization.

### Sample size

Sample size for dataset 1 was based on calculations undertaken for the larger cross-sectional study using software Epi-Info Version 3.5.2 for estimating the institutional delivery rate in rural Haryana. With power at 90%, confidence interval at 95%, expected proportion of institutional deliveries as 72% (as reported by Coverage Evaluation Survey, 2009)[10] and a design effect of 1.25 to adjust for stratification, a sample size of 90 women from each PHC area was estimated. As generally there are 4 SC under each PHC, sample size per SC area came out to be 23.

For comparison between community results and ANM record registers, we considered the use of 3 or more ANC check-ups as the comparison indicator. The baseline coverage obtained for this indicator through our community survey was around 70%. Expecting a matching within 65% to 75% range with the record registers, 80% power of the study and 95% two-sided confidence interval, we obtained a required sample size of 1291 women. However, we analysed data for 4807 women from all 209 SCs, which yielded us the power of 99.9% at state level.

### Data collection and analysis

Data for all four datasets were collected over a period of 12 months, from October 2012 to September 2013. Quality assurance measures were undertaken to prevent influence from local functionaries or investigator bias during data collection. Investigators were not allowed to make contact with ANMs and other village level health workers during household survey. Further, the investigators did not possess data from the survey at the time of collection of data from health facility. Instead they were provided with names of the selected women for whom data were to be collected from HMIS records at SC. Thus, at the time of data collection from ANM record registers, field investigators had no access to information recorded through household survey.

Data collected for seven data elements in datasets 1 and 2 and eight indicators in datasets 3 and 4 were in continuous scale or in date format. Remaining indicators were categorical in nature. Data was analysed using IBM SPSS Statistics ver. 21.

The first parameter analysed was completeness of the ANM record registers. This was defined as the proportion of women from the survey who were registered and for whom data was entered by ANM in relevant record registers. The denominator for each indicator included the total number of women covered in the household survey.

The second parameter analysed was the extent of level 1 discordance for each indicator. Two separate matching strategies, i.e. group and individual, were used to assess the adequacy of HMIS to serve the needs of programme managers and researchers. While the needs of former are usually met if the pooled coverage is accurate, researchers’ needs are for a data which is accurate at individual level. For group matching, coverage for each indicator was separately computed in datasets 1 and 2 to reveal differences in results at each SC level. Cluster robust standard errors were then generated and used for determining 95% CI for each difference at state level and its significance level. Average SC level difference was calculated as P_SC_ = ∑p_i_/c, where p_i_ was the crude difference in i^th^ SC and c was the number of clusters (SC). Standard Error was calculated as Standard Deviation/√c where Standard Deviation = √[∑(p_i_—P_SC_)^2^/c]. Confidence intervals (95%) were calculated as the mean difference at state level across clusters ± 2 SE.[11]

The datasets were next individually matched to examine the proportion of cases in which data was correctly recorded in SC record. This was termed individual matching. Different comparison windows for matching in terms of number of days were also used. This was done to accommodate needs of researchers as well as policy makers, ranging from narrow comparison windows to enhance accuracy, to broader comparison windows to account for recall bias during survey. As an example, for ‘date of delivery’ variable, comparisons were made within a window of ± 2 days to a window of ± 15 days, while the same for DPT1 administration date ranged from ± 5 days to ± 30 days.

The third parameter analysed was the extent of level 2 discordance between recorded and reported value for each indicator. For this purpose, numeric difference for each indicator in the datasets 3 and 4 was computed. All results were compared using statistical tests for assessing significance of difference between proportions at 5% alpha error.

### Ethical clearance

Ethical clearance for the study was obtained from Institute Ethics Committee of Post-Graduate Institute of Medical Education & Research, Chandigarh, India. Administrative approval was sought from National Rural Health Mission, Haryana. Written informed consent for participation was obtained from respondents in household survey and from the staff interviewed at sub-centres for accessing record data.

## Results

### Completeness of HMIS

**Fig 1:**
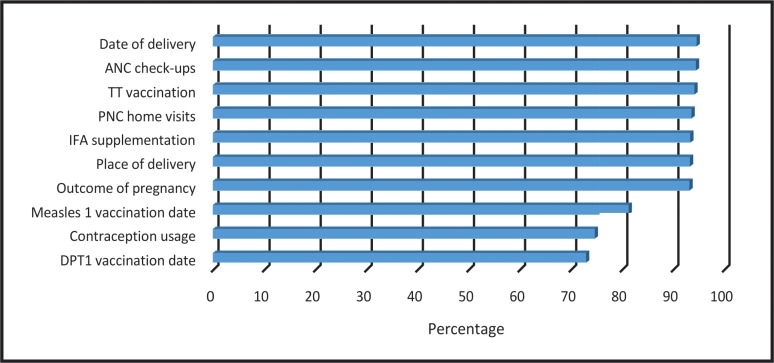
Completeness of health management information system at sub-centre level in Haryana state, India.

Completeness of ANM records ranged from 73% for ‘DPT1 vaccination date’ to 94.6% for ‘date of delivery’. Average completeness levels were found to be 88.5% with the median value being 93.3% (Fig 1).

### Quality of HMIS: Group matching

Comparison of coverage for five indicators (group matching) based on SC HMIS and household survey is presented in Table 2. Except for different categories for ‘outcome of pregnancy’, ‘TT injection coverage’ and ‘nil ANC check-ups’, all other indicators were found to have significant level 1 discordance with the HMIS record registers. The extent of level 1 discordance for ‘full IFA supplementation’ and ‘3 or more ANC visits’ was up to 41.7% and 16% respectively (Table 2).

**Table 2:** Comparison between coverage of maternal and child health services in sub-centre records and household survey in Haryana state, India.

Indicators with their categories#		Mean Sub-Centre Record Based coverage (%)	Mean Household survey coverage (%)	Mean Difference	95% CI of mean difference		p-value@
					Lower limit	Upper limit	
No. of ANC check-ups:	0	2.2	2.9	-0.7	-2.1	0.6	0.31
	1 or 2 ANC	12.8	28	-15.1	-19.7	-10.6	<0.01*
	3 or more ANC	84.3	68.5	15.8	11.2	20.5	<0.01*
No. of TT injections:	0 TT inj.	2	4.9	-2.9	-4.6	-1.3	<0.01*
	1 TT inj.	11.5	10.5	0.9	-0.9	2.6	0.34
	2 TT inj.	86.5	84.5	2.1	-0.2	4.5	0.07
No. of IFA tablets provided:	No IFA tab.	5.5	18.7	-13.1	-15.8	-10.3	<0.01*
	Less than 30 IFA tab.	0.3	7.3	-6.9	-8.1	-5.5	<0.01*
	Between 30 to 100 IFA tab.	14.4	35.9	-21.5	-24.9	-17.1	<0.01*
	100 or more IFA tab.	79.8	38.1	40.9	36.3	45.5	<0.01*
Place of delivery:	Public Sector	62.6	59.9	2.8	1.2	4.7	<0.01*
	Private Sector	24.3	26.1	-1.7	-3.1	-0.4	0.01*
	Home	12.8	13.8	-1.1	-2.2	0.1	0.06
Outcome of pregnancy:	Live Birth	99.2	99.1	0.1	-0.2	0.4	0.69
	Still Birth	0.7	0.8	-0.1	-0.4	0.2	0.46
	Abortions	0.1	0.1	0	-0.1	0.2	0.64

* Significant results

# Date of delivery and DPT 1 vaccination dates were excluded from group matching analysis due to nature of the variables.

@ Results computed using cluster robust standard errors.

### Quality of HMIS: Individual matching

Date of delivery mentioned in HMIS records matched with that reported by households in 70.5% cases, which rose to 76.5% when the window was widened to a ± 15 days period. Similarly, date of DPT1 vaccine administration mentioned in HMIS records matched with that reported by households in 65.8% cases for a ± 10 days period window and in 77.5% cases for a ± 30 days period window (Table 3).

**Table 3:** Quality of health management information system in Haryana state, India.

Indicators with their categories (Comparison window)*		Individual/ case-based matching (%)	95% Confidence Interval	
			Lower limit (%)	Upper limit (%)
Date of Delivery:	4 days matching (-2,+2)	70.5	69.1	72
	10 days matching (-5,+5)	72.2	70.8	73.7
	14 days matching (-7,+7)	73.1	71.7	74.6
	30 days matching (-15,+15)	76.5	75.2	77.9
Date of DPT1 administration:	10 days matching (-5,+5)	65.8	63.5	68.1
	20 days matching (-10,+10)	68.8	66.5	71
	30 days matching (-15,+15)	70.8	68.7	73
	60 days matching (-30,+30)	77.5	75.5	79.5
PNC visits by ANM:	Exactly same number of PNC visits recorded in register as reported by individual in community	20.1	18.8	21.4
	PNC visit recorded in register within (+1,-1) range of that obtained through community survey	43.7	42.1	45.2
	Both sources report the woman receiving at least one PNC visit	48.2	46.6	49.7
No. of ANC check-ups:	Exactly same number of ANC visits recorded in register as reported by individual in community	26.6	25.2	28.1
	Three ANC visits recorded in register where more than three ANC visits reported by individual in community	49.2	47.6	50.7
	Three ANC visits recorded in register where nil ANC visits reported by individual in community	1.2	0.8	1.5
No. of TT inj. Administered:	Exactly same number of TT injections recorded in register as reported by individual in community	81.6	80.4	82.8
	Both sources report the woman receiving at least one TT injection	93.1	92.3	93.9
No. of IFA tab. Administered:	Both sources report the woman to have received at least 90 IFA tablets	41.8	40.2	43.3
	Both sources report the woman to have received some IFA tablets (without specifying any numbers)	77	75.6	78.2
Place of delivery		89.4	88.4	90.4

* Outcome of pregnancy was excluded from individual matching analysis because of the nature of the variable.

The number of ANC visits, PNC visits and TT injections were found to be correctly recorded in HMIS in 26.6%, 20.1% and 81.6% cases respectively. In about 48.2% cases, the HMIS records and the respondents in community agreed that at least one PNC home visit was done by ANM. Figures for 3 ANC check-ups and full iron-folic acid supplementation course in HMIS records were found to be correct as per household survey findings in 49.2% and 41.8% cases respectively. Place of delivery was correctly registered in HMIS records in 89.4% cases.

### Extent of level 2 discordance

Analysis of datasets 3 and 4 for comparing ANM records with monthly reports sent to higher facilities yielded an over-reporting ranging from 1.4% to 6% for various indicators (Table 4). High levels of level 2 discordance was observed for prophylactic IFA supplementation (6% over-reporting), intra-uterine contraceptive device insertions (5.8%) and administration of TT 2^nd^ dose (5.3%).

**Table 4:** Over-reporting between sub-centre records and monthly performance reports.

	Indicator	Over-reporting %
1.	New ANC registration	1.6
2.	TT 1^st^ dose	3.0
3.	TT 2^nd^ dose	5.3
4.	Initiation of prophylactic IFA supplementation	6.0
5.	Completion of prophylactic IFA supplementation	6.0
6.	Initiation of therapeutic IFA supplementation	2.5
7.	Completion of therapeutic IFA supplementation	3.1
8.	Delivery in public sector	3.7
9.	Delivery in private sector	4.1
10.	Delivery at home	3.2
11.	DPT/ LPV 1^st^ dose	2.5
12.	Measles 1^st^ dose	1.4
13.	Vitamin A dose	3.7
14.	IUD insertions	5.8

### Results of quality assurance exercise

The results of supervisory exercises conducted by the research team yielded a difference in coverage ranging from -4.2% to 5.6% for different indicators between initial data collection by field investigators and data recollected by supervisors. (Table 5) Maximum difference was observed for proportion of beneficiaries who received two TT injections during ante-natal check-ups. These findings could be a result of recall bias, measurement error, or other factors unaccounted for at the time of data collection.

**Table 5:** Comparison between indicators coverage as per data collected by field investigators and supervisors.

S. No.	Indicator	Coverage according to investigator (%)	Coverage according to supervisor (%)	Difference in coverage (%)
1.	Nil ANC check-ups	4.23	3.15	1.1
2.	3 or more ANC check-ups	68.9	67.5	1.4
3.	2 TT injections	81.7	76.1	5.6
4.	Nil IFA tablets consumption	19.8	14.4	5.4
5.	90 or more IFA tablets consumption	36.9	41.1	-4.2
6.	3 or more PNC home visits by ANM	10.9	11.3	-0.4
7.	DPT1/ LPV1 vaccine coverage	69.6	65.4	4.2

## Discussion

Overall, HMIS records for various RCH services at Sub-Centre level in Haryana state displayed satisfactory completeness levels except for contraception records and vaccine administration dates. However quality of records was sub-optimal, with significant level 1 as well as level 2 discordance for ante-natal and post-natal services. The two most over-reported indicators at 1^st^ level were 3 or more ANC visits by pregnant woman and provision of 100 or more IFA tablets. In terms of level 2 discordance, the indicators most inflated were the ones with maximal programmatic focus and lowest actual coverage of service delivery at SC level. These included IFA supplementation, contraceptive device insertions and administration of 2^nd^ dose of TT to pregnant woman. The least over-reported were found to be those related to immunisation and pregnancy registrations. This may be due to relatively better existing vaccine coverage rates and pregnancy registrations as compared to other MCH indicators in Haryana state.

In an earlier study undertaken in Uttrakhand state in 2012, 24.6% entries were reported missing for various indicators.[12] This result was similar to our findings for DPT 1 administration date recording and contraception usage information, which lacked entered data in about 27% and 25.3% cases respectively. Our results show lower incompleteness levels for all other indicators.

Our study indicated that the variables mostly over-reported were the ones for which high levels of coverage were desired but not achieved. This finding was also observed in a previous study from Haryana where correctness of records for immunization, attendant at the time of delivery & contraception was reported to be around 66%, 60.5% & 27.5% respectively.[8, 13] Khandade et al in 2013, in their study from Bihar state, reported correctness of HMIS for various indicators to vary from 65% to 80%.[14] In our study, these figures ranged between 55% and 90% for various indicators. For number of ANC check-ups by pregnant women and number of PNC home visits by ANMs, these figures were lower than others, being between 20% and 50%. Khandade et al also reported 100% incompleteness of HMIS records for contraceptive usage in their study.[14] We found 45% incompleteness in contraceptive records (Fig 1). Another study by Harikumar in 2013 found limits of accuracy and completeness of data in Kerala state to be around 37% and 29% respectively, which are lower than results observed in our study.[15] This shows that overall quality of HMIS in Haryana was better than some other states of India, which may be attributed to efforts of the state Government towards improving quality of the same.

The major strength of our study is its large dataset which pertains to a recent period and thus portrays situation under National Rural Health Mission–India’s flagship health program. For assessment of over-reporting in ANM records, we collected and compared information for over 4800 women from more than 200 health sub-centre areas. This formed a subset of around 12% of total sub-centres in the state. No research study at such large scale for assessment of data quality and completeness has been reported in the literature earlier. The sub-centres chosen were randomly selected across all districts of Haryana and thus are geographically representative of the state.

Another major strength of this study is the use of a systematic approach for assessment of quality. None of the previous studies have attempted to assess quality of both levels of data reporting (over-recording and while report preparation) for generating a comprehensive evaluation statement. Additionally, the approach of our analysis was to serve needs of both types of audience, researchers as well as policy makers. For some of the indicators we have analysed and presented our results using different size comparison windows, where small comparison windows maintained accuracy to satisfy researchers’ inquisitiveness for precision, while progressively larger comparison windows provided flexibility to policy makers to select comparison of results as per their decision making needs. For other indicators, multiple criteria were used to generate comparisons to understand results from different point of views. We used eight and fourteen data elements for level 1 and 2 discordance, generating thirty-five and fourteen indicators to assess quality of over-recording and over-reporting respectively. The indicators may not cover entire HMIS, but do cover the most salient ones.

An important limitation of our study might be the influence of recall bias or measurement error in the results obtained from community based household survey. As 90% of data collected during quality assurance exercise matched with the original, we believe that the extent of these errors was low and unlikely to influence overall conclusion of study.

Our study is limited in terms of assessing reasons for the tendency of health workers to over-record and over-report. Some other studies attribute this tendency to inflate to a pressure from superiors to hide poor service provision at the Sub-Centre level, or to enhance the image of the facility to secure more funds. Husain and others in their study reported that ANMs accepted being pressurised to bloat figures. [12] Bhojani et al in their study from Orissa state reported inadequate supervision and accountability; and inadequate review of data before transmission to next level to be the major challenges that negatively influenced the quality of data being generated at the Sub-Centre level.[16]

Whatever the reasons for poor quality may be, policy and programme management decisions based on these sub-standard datasets can have negative consequences. Decisions are no better than the data on which they are based. Reliable, relevant, and complete data (as opposed to the incomplete data) supports organizational efficiency and is a cornerstone of sound decision-making. Thus, investing in the development of effective health information systems would enable decision-makers at all levels to monitor progress towards health goals, strengthen the evidence base for effective health policies and improve governance.[17]

This requires further research to identify and rectify the issues at grassroot level. In-depth qualitative studies can be undertaken to check motivation, knowledge & skill sets and understanding of definitions and protocols by ANMs and data entry operators who are involved in the process of data reporting. They must be made aware of the importance of different indicators in routine HMIS and their correct reporting so that genuine data can be generated for its effective use.[18] Accountability measures for ensuring correctness of data need to be institutionalised and efforts to maintain quality need to be assessed periodically so that observed deficiencies may be timely addressed.

## Supporting Information

S1 Table Data elements for assessing quality of health management information system in Haryana state, India. (DOCX)
