# Supplementary material for: Quality of Health Management Information System for Maternal & Child Health Care in Haryana State, India
Source: PLoS One. 2016 Feb 12;11(2):e0148449. doi: 10.1371/journal.pone.0148449 (PMC4752326; doi:10.1371/journal.pone.0148449)
Supplement: S1 Table — (DOCX) [file pone.0148449.s001.docx]

**S1 Table: Data elements for assessing quality of health management information system in Haryana state, India.**

**I. Indicators used for assessment of level 1 discordance:**

| S. No. | Indicator | Type of analysis involved | Background data element | Characteristic of data element | Source of information for Dataset 1 | Source of information for Dataset 2 | Numerator for indicator | Denominator for indicator |
| --- | --- | --- | --- | --- | --- | --- | --- | --- |
|  | Coverage of nil ANC check-ups | Aggregate, group level analysis, computed separately for dataset 1 and 2 | Number of ANC Check-ups received by pregnant woman during ANC | Numeric | Interview with eligible woman during household survey | Record for same woman entered in ‘ANC, PNC and immunisation’ register maintained by ANM at Sub-Centre | Total number of women who reported (in dataset 1) or who were reported (in dataset 2) to have nil ANC check-ups during ANC | Total number of women interviewed |
|  | Coverage of 1 or 2 ANC check-ups | Aggregate, group level analysis, computed separately for dataset 1 and 2 | Number of ANC Check-ups received by pregnant woman during ANC | Numeric | Interview with eligible woman during household survey | Record for same woman entered in ‘ANC, PNC and immunisation’ register maintained by ANM at Sub-Centre | Total number of women who reported (in dataset 1) or who were reported (in dataset 2) to have 1 or 2 ANC check-ups during ANC | Total number of women interviewed |
|  | Coverage of 3 or more ANC check-ups | Aggregate, group level analysis, computed separately for dataset 1 and 2 | Number of ANC Check-ups received by pregnant woman during ANC | Numeric | Interview with eligible woman during household survey | Record for same woman entered in ‘ANC, PNC and immunisation’ register maintained by ANM at Sub-Centre | Total number of women who reported (in dataset 1) or who were reported (in dataset 2) to have 3 or more ANC check-ups during ANC | Total number of women interviewed |
|  | Proportion of women reporting nil TT injections during ANC | Aggregate, group level analysis, computed separately for dataset 1 and 2 | Number of TT injections received by pregnant woman during ANC | Numeric | Interview with eligible woman during household survey | Record for same woman entered in ‘ANC, PNC and immunisation’ register maintained by ANM at Sub-Centre | Total number of women who reported (in dataset 1) or who were reported (in dataset 2) to have 0 TT injections during ANC | Total number of women interviewed |
|  | Proportion of women reporting 1 TT injection during ANC | Aggregate, group level analysis, computed separately for dataset 1 and 2 | Number of TT injections received by pregnant woman during ANC | Numeric | Interview with eligible woman during household survey | Record for same woman entered in ‘ANC, PNC and immunisation’ register maintained by ANM at Sub-Centre | Total number of women who reported (in dataset 1) or who were reported (in dataset 2) to have 1 TT injections during ANC | Total number of women interviewed |
|  | Proportion of women reporting 2 TT injections during ANC | Aggregate, group level analysis, computed separately for dataset 1 and 2 | Number of TT injections received by pregnant woman during ANC | Numeric | Interview with eligible woman during household survey | Record for same woman entered in ‘ANC, PNC and immunisation’ register maintained by ANM at Sub-Centre | Total number of women who reported (in dataset 1) or who were reported (in dataset 2) to have 2 TT injections during ANC | Total number of women interviewed |
|  | Proportion of women reporting nil IFA tablet provision during ANC | Aggregate, group level analysis, computed separately for dataset 1 and 2 | Number of IFA tablets received by pregnant woman during ANC | Numeric | Interview with eligible woman during household survey | Record for same woman entered in ‘ANC, PNC and immunisation’ register maintained by ANM at Sub-Centre | Total number of women who reported (in dataset 1) or who were reported (in dataset 2) to have received 0 IFA tablets during ANC | Total number of women interviewed |
|  | Proportion of women reporting less than 30 IFA tablet provision during ANC | Aggregate, group level analysis, computed separately for dataset 1 and 2 | Number of IFA tablets received by pregnant woman during ANC | Numeric | Interview with eligible woman during household survey | Record for same woman entered in ‘ANC, PNC and immunisation’ register maintained by ANM at Sub-Centre | Total number of women who reported (in dataset 1) or who were reported (in dataset 2) to have received more than 0 but less than 30 IFA tablets during ANC | Total number of women interviewed |
|  | Proportion of women reporting between 30 to 100 IFA tablet provision during ANC | Aggregate, group level analysis, computed separately for dataset 1 and 2 | Number of IFA tablets received by pregnant woman during ANC | Numeric | Interview with eligible woman during household survey | Record for same woman entered in ‘ANC, PNC and immunisation’ register maintained by ANM at Sub-Centre | Total number of women who reported (in dataset 1) or who were reported (in dataset 2) to have received more than 30 but less than 100 IFA tablets during ANC | Total number of women interviewed |
|  | Proportion of women reporting 100 or more IFA tablet provision during ANC | Aggregate, group level analysis, computed separately for dataset 1 and 2 | Number of IFA tablets received by pregnant woman during ANC | Numeric | Interview with eligible woman during household survey | Record for same woman entered in ‘ANC, PNC and immunisation’ register maintained by ANM at Sub-Centre | Total number of women who reported (in dataset 1) or who were reported (in dataset 2) to have received 100 or more IFA tablets during ANC | Total number of women interviewed |
|  | Proportion of public sector deliveries | Aggregate, group level analysis, computed separately for dataset 1 and 2 | Place of delivery: Public sector health facility, private health facility or home delivery | Categorical | Interview with eligible woman during household survey | Record for same woman entered in ‘ANC, PNC and immunisation’ register maintained by ANM at Sub-Centre | Total number of women who reported (in dataset 1) or who were reported (in dataset 2) to have delivered the child in public sector hospitals | Total number of women interviewed |
|  | Proportion of private sector deliveries | Aggregate, group level analysis, computed separately for dataset 1 and 2 | Place of delivery: Public sector health facility, private health facility or home delivery | Categorical | Interview with eligible woman during household survey | Record for same woman entered in ‘ANC, PNC and immunisation’ register maintained by ANM at Sub-Centre | Total number of women who reported (in dataset 1) or who were reported (in dataset 2) to have delivered the child in private sector hospitals | Total number of women interviewed |
|  | Proportion of home deliveries | Aggregate, group level analysis, computed separately for dataset 1 and 2 | Place of delivery: Public sector health facility, private health facility or home delivery | Categorical | Interview with eligible woman during household survey | Record for same woman entered in ‘ANC, PNC and immunisation’ register maintained by ANM at Sub-Centre | Total number of women who reported (in dataset 1) or who were reported (in dataset 2) to have delivered the child at home | Total number of women interviewed |
|  | Proportion of live births | Aggregate, group level analysis, computed separately for dataset 1 and 2 | Outcome of pregnancy: Live birth, still birth or abortion | Categorical | Interview with eligible woman during household survey | Record for same woman entered in ‘ANC, PNC and immunisation’ register maintained by ANM at Sub-Centre | Total number of women who reported (in dataset 1) or who were reported (in dataset 2) to have a live birth | Total number of women interviewed |
|  | Proportion of still births | Aggregate, group level analysis, computed separately for dataset 1 and 2 | Outcome of pregnancy: Live birth, still birth or abortion | Categorical | Interview with eligible woman during household survey | Record for same woman entered in ‘ANC, PNC and immunisation’ register maintained by ANM at Sub-Centre | Total number of women who reported (in dataset 1) or who were reported (in dataset 2) to have a still birth | Total number of women interviewed |
|  | Proportion of abortions | Aggregate, group level analysis, computed separately for dataset 1 and 2 | Outcome of pregnancy: Live birth, still birth or abortion | Categorical | Interview with eligible woman during household survey | Record for same woman entered in ‘ANC, PNC and immunisation’ register maintained by ANM at Sub-Centre | Total number of women who reported (in dataset 1) or who were reported (in dataset 2) to have an abortion | Total number of women interviewed |
|  | Proportion of cases with date of delivery mentioned in ANM records within ± 2 days of that reported by woman in community | Individual matching for each respondent | Date of delivery | Date | Interview with eligible woman during household survey | Record for same woman entered in ‘ANC, PNC and immunisation’ register maintained by ANM at Sub-Centre | Number of cases where date of delivery mentioned in ANM records lied within ± 2 days of that reported by woman in community | Total number of women for whom dates were matched |
|  | Proportion of cases with date of delivery mentioned in ANM records within ± 5 days of that reported by woman in community | Individual matching for each respondent | Date of delivery | Date | Interview with eligible woman during household survey | Record for same woman entered in ‘ANC, PNC and immunisation’ register maintained by ANM at Sub-Centre | Number of cases where date of delivery mentioned in ANM records lied within ± 5 days of that reported by woman in community | Total number of women for whom dates were matched |
|  | Proportion of cases with date of delivery mentioned in ANM records within ± 7 days of that reported by woman in community | Individual matching for each respondent | Date of delivery | Date | Interview with eligible woman during household survey | Record for same woman entered in ‘ANC, PNC and immunisation’ register maintained by ANM at Sub-Centre | Number of cases where date of delivery mentioned in ANM records lied within ± 7 days of that reported by woman in community | Total number of women for whom dates were matched |
|  | Proportion of cases with date of delivery mentioned in ANM records within ± 15 days of that reported by woman in community | Individual matching for each respondent | Date of delivery | Date | Interview with eligible woman during household survey | Record for same woman entered in ‘ANC, PNC and immunisation’ register maintained by ANM at Sub-Centre | Number of cases where date of delivery mentioned in ANM records lied within ± 15 days of that reported by woman in community | Total number of women for whom dates were matched |
|  | Proportion of cases with date of DPT 1 vaccine administration mentioned in ANM records within ± 5 days of that reported by woman in community | Individual matching for each respondent | DPT 1 Vaccination Date | Date | Interview with eligible woman during household survey | Record for same woman entered in ‘ANC, PNC and immunisation’ register maintained by ANM at Sub-Centre | Number of cases where date of DPT 1 vaccine administration mentioned in ANM records lied within ± 5 days of that reported by woman in community | Total number of women for whom dates were matched |
|  | Proportion of cases with date of DPT 1 vaccine administration mentioned in ANM records within ± 10 days of that reported by woman in community | Individual matching for each respondent | DPT 1 Vaccination Date | Date | Interview with eligible woman during household survey | Record for same woman entered in ‘ANC, PNC and immunisation’ register maintained by ANM at Sub-Centre | Number of cases where date of DPT 1 vaccine administration mentioned in ANM records lied within ± 10 days of that reported by woman in community | Total number of women for whom dates were matched |
|  | Proportion of cases with date of DPT 1 vaccine administration mentioned in ANM records within ± 15 days of that reported by woman in community | Individual matching for each respondent | DPT 1 Vaccination Date | Date | Interview with eligible woman during household survey | Record for same woman entered in ‘ANC, PNC and immunisation’ register maintained by ANM at Sub-Centre | Number of cases where date of DPT 1 vaccine administration mentioned in ANM records lied within ± 15 days of that reported by woman in community | Total number of women for whom dates were matched |
|  | Proportion of cases with date of DPT 1 vaccine administration mentioned in ANM records within ± 30 days of that reported by woman in community | Individual matching for each respondent | DPT 1 Vaccination Date | Date | Interview with eligible woman during household survey | Record for same woman entered in ‘ANC, PNC and immunisation’ register maintained by ANM at Sub-Centre | Number of cases where date of DPT 1 vaccine administration mentioned in ANM records lied within ± 30 days of that reported by woman in community | Total number of women for whom dates were matched |
|  | Proportion of cases where number of PNC visits mentioned in ANM records matched exactly with that reported by woman in community | Individual matching for each respondent | Number of PNC home visits conducted by ANM | Numeric | Interview with eligible woman during household survey | Record for same woman entered in ‘ANC, PNC and immunisation’ register maintained by ANM at Sub-Centre | Number of cases where number of PNC visits mentioned in ANM records matched exactly with that reported by woman in community | Total number of women for whom matching was done |
|  | Proportion of cases where number of PNC visits mentioned in ANM records were in ± 1 range of that reported by woman in community | Individual matching for each respondent | Number of PNC home visits conducted by ANM | Numeric | Interview with eligible woman during household survey | Record for same woman entered in ‘ANC, PNC and immunisation’ register maintained by ANM at Sub-Centre | Number of cases where number of PNC visits mentioned in ANM records were in ± 1 range of that reported by woman in community | Total number of women for whom matching was done |
|  | Proportion of cases where both sources mentioned at least 1 PNC visit | Individual matching for each respondent | Number of PNC home visits conducted by ANM | Numeric | Interview with eligible woman during household survey | Record for same woman entered in ‘ANC, PNC and immunisation’ register maintained by ANM at Sub-Centre | Number of cases where both sources mentioned at least 1 PNC visit | Total number of women for whom matching was done |
|  | Proportion of cases where number of ANC visits mentioned in ANM records matched exactly with that reported by woman in community | Individual matching for each respondent | Number of ANC Check-ups received by pregnant woman during ANC | Numeric | Interview with eligible woman during household survey | Record for same woman entered in ‘ANC, PNC and immunisation’ register maintained by ANM at Sub-Centre | Number of cases where number of ANC visits mentioned in ANM records matched exactly with that reported by woman in community | Total number of women for whom matching was done |
|  | Proportion of cases where both sources mentioned at least 3 ANC visits | Individual matching for each respondent | Number of ANC Check-ups received by pregnant woman during ANC | Numeric | Interview with eligible woman during household survey | Record for same woman entered in ‘ANC, PNC and immunisation’ register maintained by ANM at Sub-Centre | Number of cases where both sources mentioned at least 3 ANC visits | Total number of women for whom matching was done |
|  | Proportion of cases where ANM records mentioned 3 or more ANC visits while the same woman in community reported nil ANC visits. | Individual matching for each respondent | Number of ANC Check-ups received by pregnant woman during ANC | Numeric | Interview with eligible woman during household survey | Record for same woman entered in ‘ANC, PNC and immunisation’ register maintained by ANM at Sub-Centre | Number of cases where ANM records mentioned 3 or more ANC visits while the same woman in community reported nil ANC visits. | Total number of women for whom matching was done |
|  | Proportion of cases where number of TT injections mentioned in ANM records matched exactly with that reported by woman in community | Individual matching for each respondent | Number of TT injections received by pregnant woman during ANC | Numeric | Interview with eligible woman during household survey | Record for same woman entered in ‘ANC, PNC and immunisation’ register maintained by ANM at Sub-Centre | Number of cases where number of TT injections mentioned in ANM records matched exactly with that reported by woman in community. | Total number of women for whom matching was done |
|  | Proportion of cases where both sources mentioned the woman receiving at least 1 TT injection | Individual matching for each respondent | Number of TT injections received by pregnant woman during ANC | Numeric | Interview with eligible woman during household survey | Record for same woman entered in ‘ANC, PNC and immunisation’ register maintained by ANM at Sub-Centre | Number of cases where both sources mentioned the woman receiving at least 1 TT injection | Total number of women for whom matching was done |
|  | Proportion of cases where both sources report the woman to have received at least 90 IFA tablets during ANC | Individual matching for each respondent | Number of IFA tablets received by pregnant woman during ANC | Numeric | Interview with eligible woman during household survey | Record for same woman entered in ‘ANC, PNC and immunisation’ register maintained by ANM at Sub-Centre | Number of cases where both sources report the woman to have received at least 90 IFA tablets during ANC | Total number of women for whom matching was done |
|  | Proportion of cases where both sources report the woman to have received IFA tablets during ANC | Individual matching for each respondent | Number of IFA tablets received by pregnant woman during ANC | Numeric | Interview with eligible woman during household survey | Record for same woman entered in ‘ANC, PNC and immunisation’ register maintained by ANM at Sub-Centre | Number of cases where both sources report the woman to have received IFA tablets during ANC | Total number of women for whom matching was done |
|  | Proportion of cases where place of delivery mentioned in ANM records matched with that reported by woman in community | Individual matching for each respondent | Place of delivery: Public sector health facility, private health facility or home delivery | Categorical | Interview with eligible woman during household survey | Record for same woman entered in ‘ANC, PNC and immunisation’ register maintained by ANM at Sub-Centre | Number of cases where place of delivery mentioned in ANM records matched with that reported by woman in community | Total number of women for whom matching was done |

**II. Indicators used for assessment of level 2 discordance:**

| S. No. | Indicator | Type of analysis involved | Background data element | Characteristic of data element | Source of information for Dataset 3 | Source of information for Dataset 4 | Numerator for indicator | Denominator for indicator |
| --- | --- | --- | --- | --- | --- | --- | --- | --- |
|  | Extent of discordance between new ANC registrations reported by ANM in monthly reporting format and that recorded by ANM in relevant record register. | Aggregate, group level analysis | Number of pregnant women registered during last month | Numeric | Review of ‘ANC, PNC and immunisation’ record register maintained by ANM at Sub-Centre for last month | Report of DHIS single line reporting format submitted by ANM for the same month of reporting | Difference between new ANC registrations reported by ANM in monthly reporting format and that recorded by ANM in relevant record register for the same month. | Number of new ANC registrations recorded by ANM in relevant record register for the month |
|  | Extent of discordance between number of pregnant women who received TT1 dose as reported by ANM in monthly reporting format and that recorded by ANM in relevant record register. | Aggregate, group level analysis | Number of pregnant women who received TT1 dose | Numeric | Review of ‘ANC, PNC and immunisation’ record register maintained by ANM at Sub-Centre for last month | Report of DHIS single line reporting format submitted by ANM for the same month of reporting | Difference between number of pregnant women who received TT1 dose as reported by ANM in monthly reporting format and that recorded by ANM in relevant record register for the same month. | Number of new pregnant women who received TT1 dose as recorded by ANM in relevant record register for the month |
|  | Extent of discordance between number of pregnant women who received TT2 dose as reported by ANM in monthly reporting format and that recorded by ANM in relevant record register. | Aggregate, group level analysis | Number of pregnant women who received TT2 dose | Numeric | Review of ‘ANC, PNC and immunisation’ record register maintained by ANM at Sub-Centre for last month | Report of DHIS single line reporting format submitted by ANM for the same month of reporting | Difference between number of pregnant women who received TT2 dose as reported by ANM in monthly reporting format and that recorded by ANM in relevant record register for the same month. | Number of pregnant women who received TT2 dose as recorded by ANM in relevant record register for the month |
|  | Extent of discordance between number of pregnant women who were initiated on prophylactic IFA tablets course as reported by ANM in monthly reporting format and that recorded by ANM in relevant record register. | Aggregate, group level analysis | Number of pregnant women who were initiated on prophylactic IFA tablets course | Numeric | Review of ‘ANC, PNC and immunisation’ record register maintained by ANM at Sub-Centre for last month | Report of DHIS single line reporting format submitted by ANM for the same month of reporting | Difference between number of pregnant women who were initiated on prophylactic IFA tablets course as reported by ANM in monthly reporting format and that recorded by ANM in relevant record register for the same month. | Number of new pregnant women who were initiated on prophylactic IFA tablets course as recorded by ANM in relevant record register for the month |
|  | Extent of discordance between number of pregnant women who were initiated on therapeutic IFA tablets course as reported by ANM in monthly reporting format and that recorded by ANM in relevant record register. | Aggregate, group level analysis | Number of pregnant women who were initiated on therapeutic IFA tablets course | Numeric | Review of ‘ANC, PNC and immunisation’ record register maintained by ANM at Sub-Centre for last month | Report of DHIS single line reporting format submitted by ANM for the same month of reporting | Difference between number of pregnant women who were initiated on therapeutic IFA tablets course as reported by ANM in monthly reporting format and that recorded by ANM in relevant record register for the same month. | Number of new pregnant women who were initiated on therapeutic IFA tablets course as recorded by ANM in relevant record register for the month |
|  | Extent of discordance between number of pregnant women who completed prophylactic IFA tablets course as reported by ANM in monthly reporting format and that recorded by ANM in relevant record register. | Aggregate, group level analysis | Number of pregnant women who completed prophylactic IFA tablets consumption course | Numeric | Review of ‘ANC, PNC and immunisation’ record register maintained by ANM at Sub-Centre for last month | Report of DHIS single line reporting format submitted by ANM for the same month of reporting | Difference between number of pregnant women who completed prophylactic IFA tablets course as reported by ANM in monthly reporting format and that recorded by ANM in relevant record register for the same month. | Number of new pregnant women who completed prophylactic IFA tablets course as recorded by ANM in relevant record register for the month |
|  | Extent of discordance between number of pregnant women who completed therapeutic IFA tablets course as reported by ANM in monthly reporting format and that recorded by ANM in relevant record register. | Aggregate, group level analysis | Number of pregnant women who completed therapeutic IFA tablets consumption course | Numeric | Review of ‘ANC, PNC and immunisation’ record register maintained by ANM at Sub-Centre for last month | Report of DHIS single line reporting format submitted by ANM for the same month of reporting | Difference between number of pregnant women who completed therapeutic IFA tablets course as reported by ANM in monthly reporting format and that recorded by ANM in relevant record register for the same month. | Number of new pregnant women who completed therapeutic IFA tablets course as recorded by ANM in relevant record register for the month |
|  | Extent of discordance between public sector deliveries as reported by ANM in monthly reporting format and that recorded by her in relevant record register. | Aggregate, group level analysis | Number of women who delivered a child in public sector health facility/ private facility/ home | Numeric | Review of ‘ANC, PNC and immunisation’ record register maintained by ANM at Sub-Centre for last month | Report of DHIS single line reporting format submitted by ANM for the same month of reporting | Difference between number of public sector deliveries as reported by ANM in monthly reporting format and that recorded by her in relevant record register for the same month. | Number of public sector deliveries recorded by ANM in relevant record register for the month |
|  | Extent of discordance between private sector deliveries as reported by ANM in monthly reporting format and that recorded by her in relevant record register. | Aggregate, group level analysis | Number of women who delivered a child in public sector health facility/ private facility/ home | Numeric | Review of ‘ANC, PNC and immunisation’ record register maintained by ANM at Sub-Centre for last month | Report of DHIS single line reporting format submitted by ANM for the same month of reporting | Difference between number of private sector deliveries as reported by ANM in monthly reporting format and that recorded by her in relevant record register for the same month. | Number of private sector deliveries recorded by ANM in relevant record register for the month |
|  | Extent of discordance between home deliveries as reported by ANM in monthly reporting format and that recorded by her in relevant record register. | Aggregate, group level analysis | Number of women who delivered a child in public sector health facility/ private facility/ home | Numeric | Review of ‘ANC, PNC and immunisation’ record register maintained by ANM at Sub-Centre for last month | Report of DHIS single line reporting format submitted by ANM for the same month of reporting | Difference between number of home deliveries as reported by ANM in monthly reporting format and that recorded by her in relevant record register for the same month. | Number of home deliveries recorded by ANM in relevant record register for the month |
|  | Extent of discordance between DPT/ LPV vaccine 1^st^ doses administered as reported by ANM in monthly reporting format and that recorded by ANM in relevant record register. | Aggregate, group level analysis | Number of DPT1/ LPV1 vaccines administered | Numeric | Review of ‘ANC, PNC and immunisation’ record register maintained by ANM at Sub-Centre for last month | Report of DHIS single line reporting format submitted by ANM for the same month of reporting | Difference between number of DPT/ LPV 1^st^ doses administered as reported by ANM in monthly reporting format and that recorded by her in relevant record register for the same month. | Number of DPT/ LPV vaccine 1^st^ doses administered as recorded by ANM in relevant record register for the month |
|  | Extent of discordance between Measles vaccine 1^st^ doses administered as reported by ANM in monthly reporting format and that recorded by ANM in relevant record register. | Aggregate, group level analysis | Number of Measles 1 vaccines administered | Numeric | Review of ‘ANC, PNC and immunisation’ record register maintained by ANM at Sub-Centre for last month | Report of DHIS single line reporting format submitted by ANM for the same month of reporting | Difference between number of Measles 1^st^ doses administered as reported by ANM in monthly reporting format and that recorded by her in relevant record register for the same month. | Number of Measles vaccine 1^st^ doses administered as recorded by ANM in relevant record register for the month |
|  | Extent of discordance between Vitamin A 1^st^ doses administered as reported by ANM in monthly reporting format and that recorded by ANM in relevant record register. | Aggregate, group level analysis | Number of Vitamin A doses administered | Numeric | Review of ‘ANC, PNC and immunisation’ record register maintained by ANM at Sub-Centre for last month | Report of DHIS single line reporting format submitted by ANM for the same month of reporting | Difference between number of Vitamin A 1^st^ doses administered as reported by ANM in monthly reporting format and that recorded by her in relevant record register for the same month. | Number of Vitamin A 1^st^ doses administered as recorded by ANM in relevant record register for the month |
|  | Extent of discordance between new intra-uterine contraceptive device insertions as reported by ANM in monthly reporting format and that recorded by ANM in relevant record register. | Aggregate, group level analysis | Number of women registered under SC who received IUD insertions | Numeric | Review of ‘Family Planning’ record register maintained by ANM at Sub-Centre for last month | Report of DHIS single line reporting format submitted by ANM for the same month of reporting | Difference between number of new intra-uterine contraceptive device insertions as reported by ANM in monthly reporting format and that recorded by ANM in relevant record register for the same month. | Number of new intra-uterine contraceptive device insertions as recorded by ANM in relevant record register for the month |
